# The effects of a muscle resistance program on the functional capacity, knee extensor muscle strength and plasma levels of IL-6 and TNF-α in pre-frail elderly women: a randomized crossover clinical trial - a study protocol

**DOI:** 10.1186/1745-6215-11-82

**Published:** 2010-07-28

**Authors:** Lygia P Lustosa, Fernanda M Coelho, Juscelio P Silva, Daniele S Pereira, Adriana N Parentoni, João MD Dias, Rosangela C Dias, Leani SM Pereira

**Affiliations:** 1 Centro Universitário de Belo Horizonte, Centro Universitário Newton Paiva, Belo Horizonte, MG, Brazil; 2 Departamento de Bioquímica e Imunologia, Universidade Federal de Minas Gerais, Belo Horizonte, MG, Brazil; 3 Departamento de Fisioterapia da Universidade Federal de Minas Gerais, Belo Horizonte, MG, Brazil; 4 Departamento de Fisioterapia, Universidade Federal do Vale do Jequitinhonha e Mucuri, Diamantina, MG, Brazil

## Abstract

**Background:**

With the increase in the elderly population, a growing number of chronic degenerative diseases and a greater dependency on caregivers have been observed. Elderly persons in states of frailty remain more susceptible to significant health complications. There is evidence of an inverse relationship between plasma levels of inflammatory mediators and levels of functionality and muscle strength, suggesting that muscle-strengthening measures can aid in inflammatory conditions. The purpose of this study will be verified the effect of a muscle-strengthening program with load during a ten-week period in pre-frail elderly women with attention to the following outcomes: (1) plasma levels of interleukin-6 (IL-6) and tumor necrosis factor alpha (TNF-α), (2) functional capacity and (3) knee extensor muscle strength.

**Methods/Design:**

The study design is a randomized crossover clinical trial evaluating 26 elderly women (regardless of their race and/or social condition) who are community residents, older than 65, and classified as pre-frail according to the criteria previously described by Fried et al. (2004). All subjects will be assessed using the Timed up and go and 10-Meter Walk Test functional tests. The plasma levels of IL-6 and TNF-α will be assessed by ELISA (*enzyme-linked immunosorbent assay*) with high sensitivity kits (Quantikine^®^HS, R&D Systems Minneapolis, MN, U.S.). Knee extensor muscle strength will be assessed using the *Byodex System 3 Pro*^*® *^isokinetic dynamometer at angular speeds of 60 and 180°/s. The intervention will consist of strengthening exercises of the lower extremities at 50 to 70% of 1RM (maximal resistance) three times per week for ten weeks. The volunteers will be randomized into two groups: group E, the intervention group, and group C, the control group that did not initiate any new activities during the initial study period (ten weeks). After the initial period, group C will begin the intervention and group E will maintain everyday activities without exercising. At the end of the total study period, all volunteers will be reassessed.

**Discussion:**

To demonstrate and discuss possible influences of load-bearing exercises on the modification of plasma levels of IL-6 and TNF-α and in the functional performance of pre-frail elderly women.

**Trial Registration:**

ISRCTN62824599

## Background

Changes in the world age pyramid are occurring rapidly, such that the elderly population is projected to grow by 550,000 per year and increase to one million per year by the year 2025 [1,2]. Aging is a multifaceted reality in which genetic, biological, social, cultural and psychological variables should be considered in the differing manifestations and outcomes [3]. Frailty is a syndrome of a multifactorial nature, characterized by a reduction in energy reserves and by a decreased resistance to stressors, resulting in a cumulative decline of the physiological systems, characterized by a trio of changes: sarcopenia, neuroendocrine deregulation and immune dysfunction [4]. Fried et al. (2001) proposed five criteria for the diagnosis of frailty in the elderly [5]. Early identification of the pre-frail elderly is important in order to institute therapeutic guidelines and interventions that can possibly prevent or minimize the conditions inherent to the state of pre-frailty, such as fluctuations in health and the risk of acute clinical complications [3-5].

Current studies demonstrate that the elevation in pro-inflammatory cytokines in the plasma of the elderly is associated with the development of the frailty syndrome, a reduction in mobility, and an inability to conduct the activities of daily life, a reduction in muscle strength and an increase in mortality [6-11].

Interleukin 6 (IL-6) is an expression in plasma increases during physiological aging, probably due to the reduction of sex hormones [3,12,13]. Tumor necrosis factor alpha (TNF-α) participates in the innate acute inflammatory response by inducing the secretion of chemokines, which stimulate a second wave of cytokines, including IL-6, IL-8 and C-reactive protein [14,15]. Greiwe et al. (2001) inferred that the systematic presence of TNF-α may contribute to the loss of muscle mass [16]. There is evidence that muscular modifications appear to be associated with high levels of pro-inflammatory cytokines, especially IL-6 and TNF-α [17,18].

Regular exercise has been indicated to promoting muscle strength and functionality in the elderly and helps reduce the levels of inflammatory mediators, with a reduction in the deleterious consequences of these cytokines on muscle loss [19-22]. However, the intensity, duration of exercise and the type of muscle contraction appear to be factors that influence this outcome, though the exact mechanism is still undetermined [19,23]. There are some evidences that IL-6 could be produced from muscle contractions and that its release was dependent on the intensity of the muscle activity and the number of muscle fibers involved in the contraction, specifically the concentric and the eccentric contraction [19]. In this case it could be called miokine. They inferred that IL-6 release probably occurred through a route independent of the release of TNF-α. This cascade would result in the inhibition of the deleterious muscular effects caused by the TNF-α released systematically during sub threshold chronic inflammation [19]. Febbraio et al. (2002) suggested that the release of IL-6 could be stimulated by calcium (Ca^2+^) as a means of maintaining metabolism in homeostasis, specially in eccentric and concentric contraction [23]. However, there is still no scientific evidence concerning which mode of muscle contraction--eccentric or concentric--is optimal in promoting modifications in the plasma levels of these cytokines [19,23]. For this reason, we would like to know what happens to the new exercise program with a concentric contraction.

Some exercise programs have been proposed with the intent of minimizing the loss of muscle strength in the elderly and its functional consequences [22,24-26]. In a randomized clinical trial, Kryger et al. (2007) demonstrated earlier gains in muscle strength in elderly persons after the execution of an exercise program and there was also an increase in the transverse section area and in the percentage of type II fibers, as analyzed by a muscular biopsy [27]. However, the release of inflammatory mediators after this exercises programs is already unclear. In relation to the inflammatory mediators, Plomgaard et al. (2005) reported that IL-6 could be produced from muscle contractions and that its release was dependent on the intensity of the muscle activity and the number of muscle fibers involved in the contraction [28]. They inferred that IL-6 release probably occurred through a route independent of the release of TNF-α, inducing anti-inflammatory activity by the release of IL-1ra and IL-10.

Given this information, it can be postulated that interventions that promote the strengthening of large muscle groups may modify the plasma levels of IL-6 and TNF-α, as well as improve the activity and functional performance of elderly persons [29,30]. These modifications would be of great benefit in the prevention and treatment of disease in the elderly, since many of their issues initially manifest as a reduction in functional activity and independence. However, the literature is unanimous in pointing out that new studies should be conducted to verify the type of muscle contraction and the ideal intensity and duration of an exercise program to increase muscle strength, and to investigate its impact on the plasma levels of IL-6 and TNF-α and functionality in the elderly. The rationale is to propose kinesiotherapeutic interventions that can promote the improvement of muscle strength while decreasing the release of inflammatory mediators, sarcopenia and functional disability. Intervening in this cycle will possibly contribute towards improved functional performance of daily activities, a reduced risk of falling, improvement in the quality of life and a reduced risk of the frailty syndrome.

Thus, this randomized crossover clinical trial was proposed with the objective of verifying the effect of a program of muscle strengthening with load during a ten week period in pre-frail elderly women with attention to the following outcomes: (1) functional ability, (2) knee extensor muscle strength and (3) plasma levels of IL-6 and TNF-α. The study hypothesis is grounded in the assumption that exercising with 75% of the maximum load at a frequency of three times per week for 10 weeks may be sufficient to provide strength gains, and consequently improve functional ability and reduce the plasma levels of IL-6 and TNF-α.

This project has financial support from the Conselho Nacional de Desenvolvimento Científico e Tecnológico - CNPq, Brasília, Brasil.

Clinical Trial Registration: ISRCTN62824599.

## Methods/Design

The general objective of the study will be verified the effect of a program of resistance exercises at 75% RM on the functional capacity, knee extensor muscle strength, and to measure the plasma levels of IL-6 and sTNFr in pre-frail elderly women.

Questions/Hypotheses

1. Among pre-frail elderly women, will changes occur in the plasma levels of the inflammatory mediators IL-6 and sTNFr, functional capacity and knee extensor muscle strength after completing a program of resistance exercises at 75% of 1RM, three times a week, for 10 weeks?

2. In a follow-up three months after finishing the training, will there be modifications in the functional capacity, knee extensor muscle strength and the plasma levels of IL-6 and sTNFr in pre-frail elderly women?

3. Is there a correlation between functional capacity, knee extensor muscle strength and plasma levels of IL-6 and sTNFr before and after a training program with load in pre-frail elderly women?

**Figure 1:**
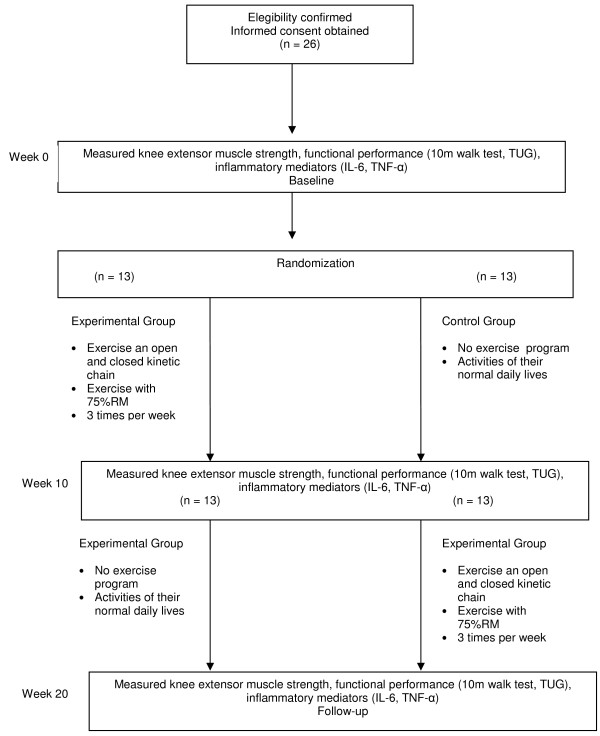


The study design is a blind randomized crossover clinical trial. Participants will be recruited from two universities. An initial assessment will be undertaken after recruitment and prior to randomization into either experimental or control group. Participants in the experimental group will receive an exercise program, three times a week, during ten weeks. Participants in the control group will receive no physiotherapy intervention. Outcomes will be measured again after ten weeks to reflect the immediate effect of the intervention. Then, the experimental group will receive no physiotherapy intervention and the control group will receive the same exercise program. Outcomes will be measured again to reflect longer-term outcomes from experimental group and the immediate effect of the intervention in the control group (Figure 1). This allows verification of the existence of a change and/or variable correlations specifically due to the intervention, and not due to the time factor and/or changes in the aging process. Furthermore, it allows verification of the existence of variable changes after finishing the training. Additionally, from an ethical point of view, it guarantees that the volunteers receive the same treatment. The outcome measures will be collected by a physiotherapist who was blind to group allocation. The responsible for the intervention will not know about the assessment. The study was approved by the ethical committee of the university and informed consent procedures were taken.

### Sample

Twenty-six women, regardless of their race and/or social class, above the age of 65 who are community residents and classified as pre-frail according to the criteria established by Fried et al. (2001) will be selected for inclusion in the study. These volunteers will be randomly divided into two groups (control = C and experimental = E) of 13 volunteers each. This population was chosen based upon the assumption that pre-frail elderly people appear to respond better to interventions, resulting in a greater possibility of modifications that influence muscle response.^3 ^Demographic characteristics (age, race, school level, height, weight and body mass index) will be collected from all the participants.

Elderly women who meet one or more of the following criteria will be excluded from the study: previous lower extremities orthopedic surgery, a history of fractures within the past year, an inability to walk unaided, carriers of neurological diseases, diagnosed acute inflammatory disease that could interfere in the assessments and the program, tumor growth in the last five years, current use of immunomodulating medications and cognitive impairment based on the mini-mental state examination [31].

### Primary outcome

Plasma levels of IL-6 and TNF-α will be determined using the ELISA (*enzyme-linked immuno sorbent assay*) method with high sensitivity kits (Quantikine^®^HS, R&D Systems Minneapolis, USA). Samples will be read with an adjusted microplate reader for 490 nm and wavelength correction to 650 nm. TNF-α acts at two known receptors: TNFR1 and TNFR2. In humans, the measure of circulating levels of the two soluble receptors is useful in determining the global production of TNF-α in plasma [32].

### Secondary outcomes

For functional performance, the Timed Up and Go (TUG) test [25,30,33] and the 10-Meter Walk Test (10MWT) will be used [34]. The TUG test has a high test-retest and inter-examiner reliability (ICC = 0.99, r = 0.93) [35]. Skinkai (2000) asserted that the 10MWT is the greatest predictor of falls and functional dependency in the elderly [34].

Knee extensor muscle strength will be evaluated with the Biodex system 3 *Pro*^*® *^isokinetic dynamometer at angular speeds of 60 and 180°/s. This equipment is capable of evaluating muscle function in concentric, eccentric and isometric modes at angular speeds of 2 to 420°/s. It will be used to measure the maximum work produced by the knee extensor muscle group normalized to body weight in the concentric and at angular speeds of 60 and 180°/s. Peak torque and potential at these speeds will also be assessed. The advantages of using an isokinetic dynamometer include using the same resistance at one constant speed of motion, a high reliability in the measures and a correlation between the torque, work and potential parameters (ICC = 0.99, p < 0.01) [35].

### Randomization

After the initial evaluation, the volunteers will be randomized into either group E or group C. This randomization will take place with an equal number of envelopes. The researcher responsible for the evaluations will be blinded to the participants' group. The researcher responsible for the intervention will be blinded to the assessment results until the final data collection.

### Intervention

The volunteers in experimental group will begin the physical activity program of exercises conducted in small groups with direct instruction from the physical therapist for one hour, three times per week for a period of ten weeks. The large muscle groups of the lower extremities (hip flexors and extensors, knee flexors and extensors and plantar flexors) will be exercised on an open and closed kinetic chain. The appropriate weight for each volunteer will be determined by calculating the percent of maximum load (1RM) [25]. The choice of concentric exercises was based upon literature which has indicated that this activity in the large muscle groups induces and modifies the production of IL-6 and TNF-α [3,12,19,28]. Semi-squats are used for their increased functional benefits and because they decrease the compression forces on the patellofemoral joint, thereby minimizing the risk of pain [22]. The session will begin with ten minutes of walking as a warm-up. Next, hip flexion, extension, abduction and adduction and knee flexion and extension motions will be conducted in an open kinematic chain, along with a semi-squat, aimed at strengthening the muscles of the lower extremities [25]. Hip exercises will be conducted in the supine (hip flexion and adduction), lateral (hip abduction) and prone (hip extension and knee flexion) positions. The semi-squat exercises will be conducted while standing with up to 60 degrees of knee flexion and with bilateral support. The concentric knee extension exercises will be conducted with the volunteer seated. The feet remained on the floor, maintaining an initial knee angle of 70 degrees of flexion. They will be instructed to extend the knee from 70 to 20 degrees of flexion, without terminally extending the knee. The program will consist of a total of 30 one-hour sessions, conducted three times per week over ten weeks. At the end of each training day, relaxation and cool-down exercises will be performed.

The volunteers in control group will be instructed to refrain from beginning any type of new physical activity for a period of the study, maintaining the activities of their normal daily lives.

After ten weeks, all the volunteers will be reevaluated with the same tests and parameters conducted in the first assessment. After this reevaluation, the groups will switch and those women who had completed the exercise program (experimental group) will now remain inactive. Those women who had previously been inactive (control group) will begin the same resistance program the experimental group had performed (Figure 1).

### RM calculation

In the first session, the maximum resistance test (RM) will be conducted for each volunteer using the 1RM test for knee extension and flexion. For the knee extension test, the volunteer will be asked to sit on a standard stretcher, in the same manner that was previously described for the concentric knee extension exercise. One leg weight of 1 kg will be placed on the ankle of the dominant leg, and the volunteer will be instructed to extend the knee fully, without changing or increasing the speed of the motion, from the starting position of 90 degrees of flexion with the foot on the floor. A rest period of five minutes will be given and the motion will then be repeated with an increase of 1 or 2 kg, according to the degree of difficulty reported by the volunteer. The maximum load will be considered to be the weight at which the volunteer is unable to perform the motion to its fullest extent, shows signs of fatigue and/or increases the speed of the motion, with no more than five attempts made throughout the day. With the RM established for knee extension, the work percentage will be calculated. In the first six sessions, 50% of 1RM will be used. Beginning with the seventh session, the load used will be increased to 75% of 1RM [25]. Every two weeks, or every six sessions, a new 1RM test will be conducted to adjust the load. Three sets of eight repetitions for each one of the exercises will be standardized in relation to the workload, as described in previous studies [25].

For calculating the 1RM for the knee flexors, the volunteer will be placed in the prone position and instructed to flex the knee from 0 degrees to 45 degrees. The same parameters as previously described in relation to the load, interval and observation of the executed motion will be considered in order to establish the RM for the knee flexors. The same percentage of 50% and 75% of 1RM will be used for the work.

### Procedures

Initially, the elderly women will answer a questionnaire for inclusion in the study and to characterize the study sample. Next, the elderly women were characterized as frail, pre-frail or non-frail according to the criteria proposed by Fried et al. (2001). Only those women classified as pre-frail will be invited to participate in the training program. All participants will undergo the TUG test [25,30,33], the 10MWT [34] and the knee extensor muscle strength test. Participants will also be tested with the isokinetic dynamometer at angular speeds of 60 and 180°/s and the plasma levels of IL-6 and TNF-α measured. All women will wear their everyday shoes or those that they consider the most comfortable.

For conducting the TUG test, the volunteer will be instructed to sit in a standard chair 45 cm in height with arm supports at a height of 65 cm (using the floor as a reference). At the verbal command "Are you ready? Go" the participant will stand, walk three meters (previously marked on the floor), turn, return to the chair and sit down again. A walking chronometer (*Q & Q, Japan CBM Corp*) will begin timing the moment the volunteer's trunk no longer touches the back of the chair, and will stop when the volunteer's trunk returns to leaning against the back of the chair. The displacement time in seconds will be noted for analysis.

Next, the 10MWT will be conducted. The volunteer will walk a course of ten meters (marked on a level floor), with two meters for acceleration and two meters for deceleration. The verbal command to begin will be the same as that used for the TUG test. The chronometer will begin as the volunteer passes the first two acceleration meters and will stop when the ten meters of the course are completed. The displacement time in seconds will be used for analysis. The volunteer will be instructed to walk at her normal pace.

To evaluate the knee extensor muscle strength, the volunteer will be positioned in the Biodex System 3 Pro^*® *^dynamometer chair. Her trunk will remain supported at 80° of hip flexion and will be secured with equipment positioning belts. She will be instructed to exert a maximum force and will be encouraged with handclaps and phrases such as "Let's go! Great. Don't stop." For each speed there will be a practice drill with three repetitions for familiarization. The test will be conducted by measuring five repetitions at an angular speed of 60°/s and fifteen repetitions an angular speeds of 180°/s, all at maximal effort, according to the assessment protocol that has previously been described [36]. The variables analyzed were the percentage of work normalized to body weight, the peak torque in Newton per meter (N/m) and the potential in watts (W).

The analysis of the plasma concentrations of IL-6 and TNF-α will be conducted in a laboratory on a different day than the functional and muscle strength measurements. These exams will be conducted in the morning, between 8:00 and 10:00 a.m., to guarantee that the circadian rhythm does not influence the measurements [37,38]. The volunteer will be instructed to eat normally the day before. A qualified professional will collect the blood, following the necessary patterns and procedures. Five milliliters of the collected blood will be centrifuged for 15 minutes and the plasma will be stored in a properly identified freezer (-70°C). Subsequently, duplicate analyses will be conducted using an ELISA with high sensitivity kits (Quantikine^®^HS, R&D Systems Minneapolis, USA) in accordance with the manufacturer's instructions and the analysis protocol. The results will be presented as the mean ± standard deviation.

### Sample size

The sample size was calculated after a previous pilot study with 12 participants, considering a confidence interval of 95%, a bilateral hypothesis test, a significance level of 0.05 (type I error), a power of 80% (type II error) and a crossover design for a statistical analysis of ANOVA. First, we calculated the size effect (f) for the partial ANOVA for the 12 participants, according to Portner & Watkins [39]. We use the functional performance (time in seconds of the TUG). We considered two freedom degrees and a power of 80%. It was determined that, considering a moderate effect size (0.5) for a minimum clinically significant difference, 13 volunteers will be needed in each group.

### Statistical analysis

The statistical analysis will be conducted using SPSS 15.0 in Windows. In order to verify data normality, the Shapiro-Wilk test will be used. A comparison of the variables of muscle strength, functional capacity and plasma levels of IL-6 and TNF-α, before and after the intervention and three months after the completed intervention, will be conducted using ANOVA. A post hoc analysis will be conducted to find possible differences. A correlation among the variables will be analyzed using the test of Pearson's correlation. An association among the variables of IL-6 and TNF-α plasma levels, functional capacity and muscle strength will be analyzed using a linear regression. The significance level will be p = 0.05.

## Conclusions

In an attempt to better clarify the role of inflammatory mediators in the aging process, our group previously demonstrated the association among inflammatory mediators and functional outcomes and muscle strength in elderly community residents and inpatients [39,40]. Similarly, the literature provides evidence of IL-6 release after strenuous exercise in elderly persons and in youth. This release may bring a benefit by decreasing TNF-α levels [3,6-8,10,12,13]. However, the degree of intensity and the activity load, as well as the mechanism of this phenomenon, are not yet established. This trial was designed in order to be reproducible in a research environment and the clinical setting. We expect to collect results at the end of July 2010. We anticipate that the results will have implications in the development of new treatment strategies for elderly persons and the modification of their frail conditions.

## Competing interests

The authors declare that they have no competing interests.

## Authors' contributions

LPL and LSMP were responsible for the conception and implementation of the study, as well as for its writing and final corrections. JMDD, RCD and ANP helped in the implementation and supervision of the study. LPL and DSP were responsible for supervision of the intervention. FMC and JPS were responsible for the evaluations. LPL, LSMP, RCD and ANP conducted the data analysis. All the authors read and approved the final manuscript.
